# Immunotherapy – 2076. A controlled study of delta inulin-adjuvanted honey bee venom immunotherapy

**DOI:** 10.1186/1939-4551-6-S1-P158

**Published:** 2013-04-23

**Authors:** Robert Heddle, Paul Russo, Nikolai Petrovsky, Rory Hanna, Anthony Smith

**Affiliations:** 1 Royal Adelaide Hospital, Flinders University, Adelaide; 2 Royal Adelaide Hospital, Australia; 3 Flinders University, Vaxine Pty Ltd, Australia; 4 Royal Adelaide Hospital, Australia; 5 Flinders Medical Centre, Australia

## Background

Honey bee (HB) venom immunotherapy (HBVIT) reduces the frequency of immediate generalised reactions (IGR) to subsequent sting by only ~ 80% and in the process induces IGR in many subjects. In influenza vaccine studies delta inulin adjuvant enhanced immunogenicity and provided antigen-sparing without any adverse reactions. In an ongoing double-blinded clinical trial we are studying the benefits of inulin adjuvant for HBVIT.

## Methods

Following institutional ethics committee approval, 26 subjects with a history of IGR to HB sting were randomized 2:1 to receive Albey (Stallergenes) HBVIT (100 mcg maintenance) by clustered, semi-rush regime with (gp A) or without (gp B) Advax™ inulin adjuvant (Vaxine Pty Ltd). Specific IgE (sIgE) was measured by CAP, and specific IgG1 and IgG4 by ELISA.

## Results

Clinicians remain blinded to patient randomization. Two subjects have withdrawn for personal reasons. One subject had two anaphylactic reactions at 3 and 100ug of venom, was withdrawn and on breaking code was in gp B (no adjuvant). Two other subjects had mild systemic reactions. A major difference between groups is apparent in sIgG4 responses. Both groups showed a peak sIgG4 response at 14 weeks (early maintenance HBVIT). In gp A however, the sIgG4 rise started earlier, the peak response was much higher and after 12 months of maintenance HBVIT, sIgG4 levels were ~3 fold higher by ELISA OD [results mean (SEM); **baseline** gp A 0.110 (0.032),gp B 0.076 (0.038), **peak** .gp A .0.822 (0.155), gp B 0.326 ((0.106), **52 weeks** gp A 0.453 (0.223) gp B 0.170 (0.059)]. sIgE responses showed a wide scatter with a rise from baseline of similar magnitude but occurring earlier in group A, followed by a progressive fall, [ **baseline** gp A 0.960 ((0.171), gp B 0.624 (0.138), **peak** gp A. 1.242 (0.154), gp B 0.888 (0.163), **52 weeks** gp A 0.862 (0.243), gp B 0.541 (0.117)]. sIgG1 responses showed a similar pattern.

## Conclusions

With the caveat that only surrogate markers have yet been analysed, delta inulin adjuvant appears to enhance the immunogenicity of HBVIT and to favour sIgG4 responses.

